# Awareness of Dental Outreach Camps and Perceptions Toward Mobile Phone-Based Communication Among Dental Outreach Camp Attendees in Chennai: A Cross-Sectional Study

**DOI:** 10.7759/cureus.106558

**Published:** 2026-04-06

**Authors:** Nemi Sri K, Narayanasamy Krishnasamy, Kumara Raja B, Madankumar Parangimalai Diwakar, Kalpana Singaram, Anitha D

**Affiliations:** 1 Public Health Dentistry, Ragas Dental College and Hospital, Chennai, IND; 2 Gastroenterology, The Tamil Nadu Dr. M.G.R. Medical University, Chennai, IND; 3 Epidemiology and Public Health, The Tamil Nadu Dr. M.G.R. Medical University, Chennai, IND; 4 Research, The Tamil Nadu Dr. M.G.R. Medical University, Chennai, IND

**Keywords:** accessibility, communication in healthcare, dentistry, mhealth (mobile health), outreach programs, perception levels, public health awareness

## Abstract

Background: Dental outreach camps are an important public health strategy for improving access to oral healthcare; however, their effectiveness depends on timely awareness and effective communication. Evidence on patients’ awareness pathways and acceptance of mobile phone-based communication in urban and semi-urban settings remains limited.

Objectives: This study aimed to (1) assess the sources and timing of awareness of dental outreach camps among attendees; (2) evaluate advance awareness of services provided; (3) assess expectations regarding services offered; (4) evaluate perceptions and willingness toward mobile phone-based communication for outreach information, reminders, and registration; and (5) examine, as a secondary objective, the association between age group and willingness toward mobile phone-based communication among outreach camp attendees in Chennai.

Methods: A cross-sectional questionnaire-based study was conducted among 250 adults attending five dental outreach camps in rural and semi-urban areas of Chennai between September 2025 and October 2025. Data were collected using a QR code-based electronic survey. Descriptive statistics and Chi-square/Fisher’s exact tests were used, with statistical significance set at p < 0.05.

Results: Word of mouth was the predominant source of awareness (66.8%), with 67.2% of participants learning about the camp on the same day. Only 36.0% were aware of the services offered in advance. Among smartphone-owning participants, acceptance of mobile phone-based communication was high for receiving information (75.6%), reminders (74.8%), registration (72.0%), and locating camps (76.0%), though these figures may overestimate acceptance in the broader outreach population given the smartphone-based recruitment method. Younger age groups showed significantly greater willingness toward mobile-based communication (p < 0.05).

Conclusion: Despite predominantly last-minute interpersonal awareness, high acceptance of mobile phone-based communication among smartphone-owning attendees suggests potential for integrating digital strategies with traditional community-based approaches to strengthen dental outreach programs, pending broader population-level validation.

## Introduction

Oral health is an integral component of general health and well-being; however, it continues to be one of the most neglected areas of healthcare globally. According to the World Health Organization (WHO), approximately 3.5 billion people worldwide are affected by oral diseases, making them among the most prevalent noncommunicable diseases (NCDs) [1]. Untreated dental caries affects nearly 2.5 billion individuals, while severe periodontal disease impacts close to 1 billion people worldwide and remains a major cause of tooth loss [1]. These oral conditions significantly impair quality of life, nutritional intake, speech, self-esteem, and productivity, thereby contributing substantially to the social and economic burden of disease, particularly in low- and middle-income countries [1].

Despite advancements in dental science and increased availability of dental professionals, access to oral healthcare remains uneven and inequitable. High treatment costs, lack of insurance coverage, geographic inaccessibility, and inadequate integration of oral health services into primary healthcare systems continue to limit utilization, especially among vulnerable populations such as rural residents, older adults, and socioeconomically disadvantaged groups [1]. In India, these challenges are further compounded by regional disparities, uneven distribution of dental manpower, and a predominantly clinic-based, curative care model [2].

Several Indian studies have consistently documented barriers to dental service utilization. A community-based survey conducted in Udaipur District, Rajasthan, reported treatment cost, dental fear, lack of perceived need, and distance to dental clinics as the most common deterrents to seeking dental care, with younger and more educated individuals demonstrating higher service utilization [3]. A meta-analysis of studies conducted across India between 2011 and 2022 revealed that only 23.96% of the adult population utilized dental care services, indicating a wide gap between disease burden and service uptake [4].

To address inequities in access, dental outreach camps have emerged as an important public health strategy, particularly for underserved and hard-to-reach populations. Outreach camps aim to deliver preventive, promotive, and basic curative dental services directly within communities, thereby reducing financial and geographic barriers [2]. Poor dissemination of information has been shown to result in delayed attendance, unmet expectations, and underutilization of available services [5]. In addition to service delivery, such camps provide opportunities for oral health education, early detection of disease, and referral for advanced care [6]. However, the effectiveness of outreach programs is strongly influenced by timely awareness, clarity of information regarding services offered, and ease of access to camp locations.

Notably, even in urban and semi-urban areas with relatively higher literacy levels and healthcare availability, advanced awareness of dental outreach camps often remains suboptimal [5]. This suggests inefficiencies in conventional communication strategies such as word-of-mouth dissemination, posters, and local announcements [3,5]. In rapidly urbanizing cities like Chennai, competing work schedules, traffic congestion, and fragmented community networks further challenge effective information dissemination, limiting the reach and impact of outreach activities [4].

The rapid expansion of mobile phone and smartphone usage in India offers a promising avenue to address these communication gaps. Mobile-based communication strategies, including text messages, phone calls, social media platforms, QR-based registrations, and mobile applications, have demonstrated effectiveness in improving healthcare awareness, appointment adherence, and patient engagement across various public health programs [7-9]. However, acceptance of digital health interventions varies across age groups, with older adults often demonstrating lower uptake due to technology hesitancy and digital divides [10].

Although mobile health interventions are increasingly adopted, evidence regarding patients' awareness pathways and willingness to receive dental outreach-related information through mobile phone-based communication remains limited, particularly in the context of dental outreach camps conducted in urban and semi-urban populations [7,8]. Furthermore, limited data are available on age-wise differences in acceptance of mobile-based communication for outreach-related information, reminders, and registration [10].

Understanding how patients become aware of dental outreach camps, the timing and clarity of information received, and their perceptions toward mobile phone-based communication is essential for optimizing outreach planning and improving service utilization. Such evidence is crucial for developing hybrid outreach models that integrate traditional community-based approaches with digital communication strategies, as emphasized in national oral health initiatives and policy frameworks [11,12]. Long-term evaluations have demonstrated that well-planned outreach programs supported by effective communication strategies can significantly improve service utilization and preventive care uptake [13].

Therefore, the present study was undertaken with the following objectives: to assess the sources and timing of awareness of dental outreach camps among attendees in Chennai, to evaluate participants' advanced awareness of services provided at outreach camps, to assess participants' expectations regarding services offered during dental outreach camps, to evaluate perceptions and willingness toward mobile phone-based communication for receiving outreach-related information, reminders, and registration details.

The following is the secondary objective: to examine the association between age group and willingness toward mobile phone-based communication among outreach camp attendees in Chennai, in order to inform evidence-based strategies for strengthening dental outreach programs in urban and semi-urban settings.

## Materials and methods

Study design

A cross-sectional questionnaire-based study was conducted to assess patients' awareness of dental outreach camps, with specific emphasis on how participants became aware of the camps and the timing and mode of information received, and to evaluate their perception of receiving dental outreach camp-related information through mobile phone-based communication.

Study setting and duration

The study was carried out during five dental outreach camps organized by Ragas Dental College and Hospital at selected rural and semi-urban community locations across Chennai, Tamil Nadu. Data collection was undertaken over a period of two months, from September 2025 to October 2025.

Sample size calculation

The sample size was calculated using G*Power software (version 3.1.9.7) [14] based on a previous study by Talukdar et al. [4]. Assuming a medium effect size (w = 0.3), a significance level (α) of 0.05, and a power of 80%, the minimum required sample size was estimated to be 248 participants. To ensure adequacy of statistical power and completeness of analysis, all eligible participants who met the inclusion criteria during the study period were considered, resulting in a final sample size of 250 participants.

Study population

The study population comprised adult individuals attending the dental outreach camps. Of the 368 individuals who attended the camps, 69 were unable to participate due to a lack of smartphone access or difficulty operating the QR code-based survey and were therefore excluded prior to enrolment. Of the remaining eligible attendees, 278 consented to participate in the study. After further exclusion of incomplete and duplicate responses, a total of 250 participants were included in the final analysis. Both first-time and repeat attendees who availed preventive or basic dental services during the dental outreach camps were included.

Ethical considerations

This prospective cross-sectional study involving human participants was reviewed and approved by the Institutional Ethics Committee of Ragas Dental College and Hospital, Chennai, Tamil Nadu, India (approval no: RIEC/20250827/PHD; date of approval: August 13, 2025). All procedures performed were in accordance with the ethical standards of the institutional research committee and with the 1964 Declaration of Helsinki and its later amendments. Written informed consent was obtained from all participants prior to enrolment.

Eligibility criteria

Inclusion Criteria

Individuals aged 18 years and above who were literate in either Tamil or English were included in the study. Participants were required to be smartphone users capable of accessing a QR code-based survey and willing to provide informed consent to participate.

Exclusion Criteria

Individuals who were unable to provide informed consent due to cognitive or physical impairments were excluded from the study. Participants who could not read either Tamil or English, as well as those who submitted incomplete, duplicate, or partially filled questionnaires, were also excluded.

Study tool

An electronic survey (e-survey) was developed using Google Forms to assess patients' awareness of and accessibility to dental outreach camps. The questionnaire consisted of 15 items organized into four domains: sociodemographic characteristics, awareness of dental outreach camps, perceptions toward mobile phone-based communication, and expectations regarding services provided during outreach programs. The sociodemographic section included questions related to age, gender, educational qualification, and occupation. The awareness domain assessed how participants first learned about the dental outreach camps and the timing of the information received. The perception domain evaluated participants' willingness to receive dental outreach camp information through mobile phone-based communication, including reminders, registration services, location tracking of nearby camps, and perceived usefulness of such communication strategies. The final domain assessed participants' expectations regarding services provided at dental outreach camps, including dental check-ups, scaling and cleaning, treatment services, and oral health education.

The questionnaire used in this study was self-developed by the investigators based on a review of existing literature and public health communication frameworks. No proprietary scales or copyrighted instruments were used; therefore, no licensing permission was required. Google Forms was utilized as a free data collection platform in accordance with its public usage policy. The questionnaire consisted of multiple-choice and dichotomous (yes/no) response formats. Content validity was assessed using Lawshe's Content Validity Ratio (CVR) method [15]. The instrument was evaluated by three experts in public health dentistry, yielding a CVR of 0.84, which indicates good content validity. The questionnaire was pretested among 20 individuals representative of the study population to assess clarity, language comprehension, and feasibility. The complete questionnaire, inclusive of all 15 items across the four domains, is provided as Supplementary File 1.

Data collection

Data were collected using a QR code-based digital survey system. The QR code was displayed at the registration desk of each dental outreach camp. Upon scanning the QR code, the questionnaire automatically appeared in Tamil or English, depending on the language settings of the participant's mobile phone, facilitating ease of comprehension and participation. Participants completed the questionnaire independently using their smartphones. For participants who required assistance with scanning or navigation, trained volunteers provided support without influencing responses.

Data analysis

Data were coded and entered into MS Excel (Microsoft Corporation, Redmond, Washington, United States) and subsequently analyzed using the IBM SPSS Statistics for Windows, Version 22 (Released 2013; IBM Corp., Armonk, New York, United States). Descriptive statistics, including frequencies and percentages, were used to summarize sociodemographic characteristics, awareness of dental outreach camps, and perceptions toward mobile phone-based communication.

The association between age groups and willingness toward mobile phone-based communication for dental outreach camps was assessed using the Chi-square test. Fisher's exact test was applied in instances where any expected cell count was less than 5. A p-value of <0.05 was considered statistically significant.

## Results

A total of 250 participants completed the survey during the two-month dental outreach camp period. The results are presented in relation to the study objectives and are organized under sociodemographic characteristics, awareness of dental outreach programs, perceptions toward mobile phone-based communication, expectations regarding dental camp services, and age-wise associations.

The sociodemographic characteristics of the participants are summarized in Table 1. The majority of participants belonged to the 26-40 years age group (43.2%), followed by those aged 18-25 years (29.6%), 41-60 years (20.0%), and above 60 years (7.2%). Females constituted 49.6% of the study population, while 46.8% were males, and 3.6% preferred not to disclose their gender. Regarding educational status, most participants had completed graduate-level education (30.0%) or secondary education (29.6%). Students (20.4%) and private employees (18.8%) were the most common occupational categories.

**Table 1:** Sociodemographic characteristics of the study participants (n = 250)

Variable	Category	n (%)
Age (years)	18-25	74 (29.6)
	26-40	108 (43.2)
	41-60	50 (20.0)
	>60	18 (7.2)
Gender	Male	117 (46.8)
	Female	124 (49.6)
	Prefer not to say	9 (3.6)
Education	No formal education	26 (10.4)
	Primary school	37 (14.8)
	Secondary school	74 (29.6)
	Graduate	75 (30.0)
	Postgraduate & above	38 (15.2)
Occupation	Unemployed	10 (4.0)
	Daily wage worker	25 (10.0)
	Self-employed	34 (13.6)
	Private employee	47 (18.8)
	Government employee	28 (11.2)
	Student	51 (20.4)
	Homemaker	38 (15.2)
	Other	17 (6.8)

**Figure 1:**
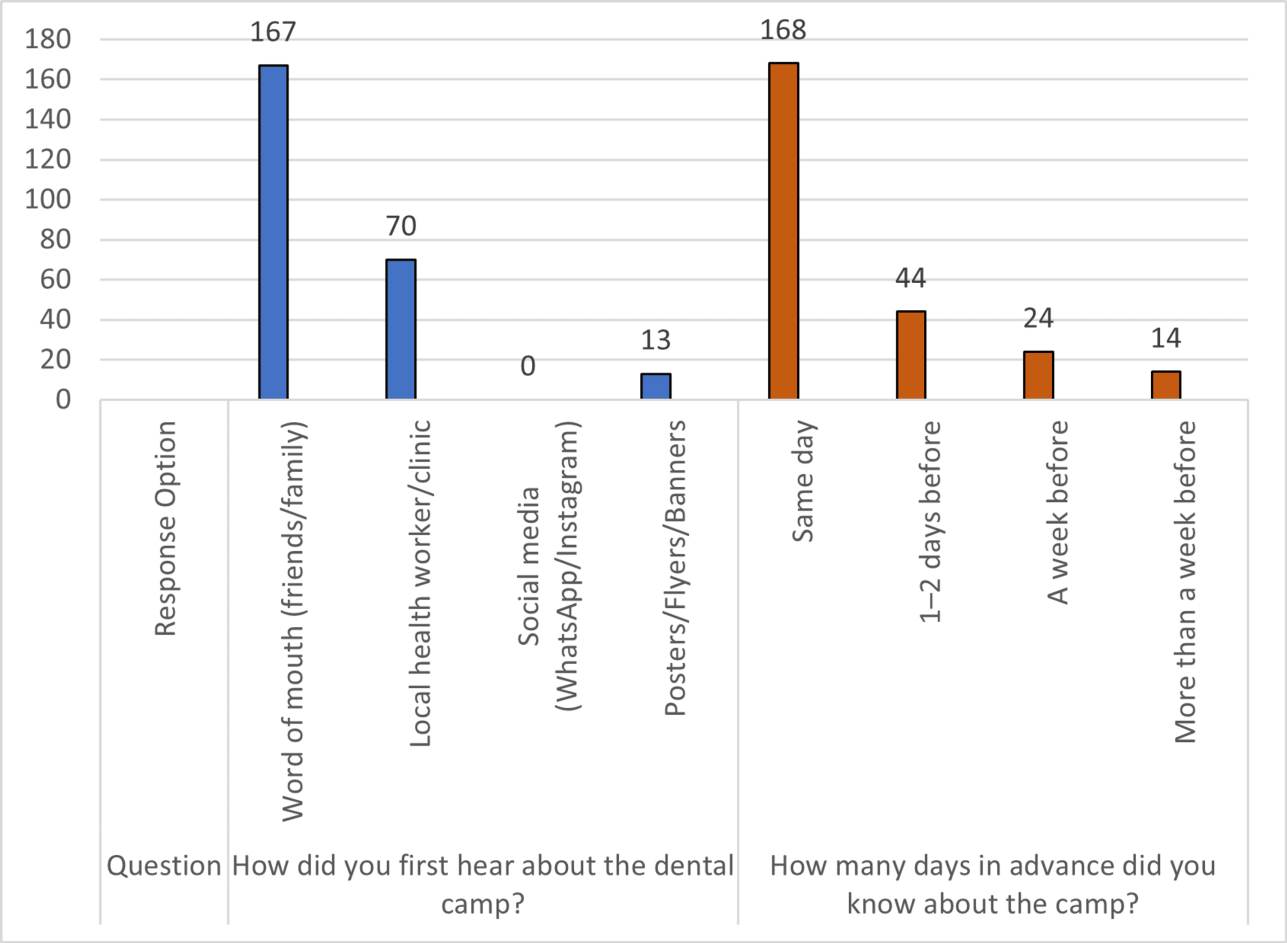
Awareness regarding dental outreach programs, including source of information and timing of awareness, among study participants (n = 250)

**Figure 2:**
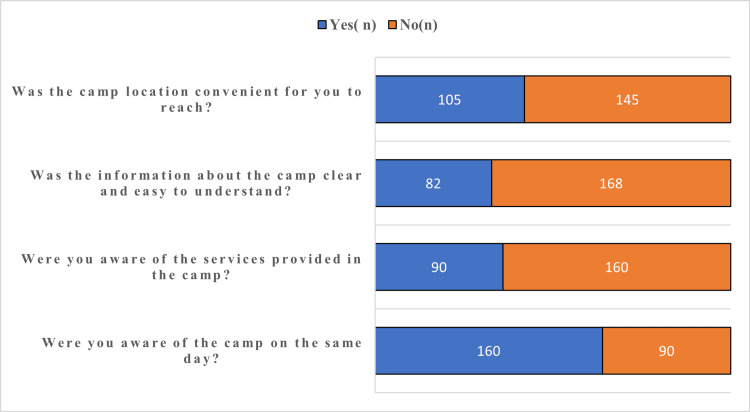
Awareness, access, and understanding of the dental outreach program among study participants (n = 250)

With respect to awareness of dental outreach programs, word of mouth was the predominant source of information (66.8%), followed by local health workers or clinics (28.0%) (Figure 1). A majority of participants (64.0%) became aware of the dental outreach camp on the same day, while 17.6% received information 1-2 days prior, 9.6% a week before, and 5.6% more than a week in advance. Regarding access and understanding of the outreach program (Figure 2), 64.0% of participants reported being aware of the camp only on the same day. Awareness of the services provided prior to attendance was reported by 36.0% of participants, while 32.8% felt that the information provided was clear and easy to understand. Additionally, 42.0% perceived the camp location as convenient to reach.

**Figure 3:**
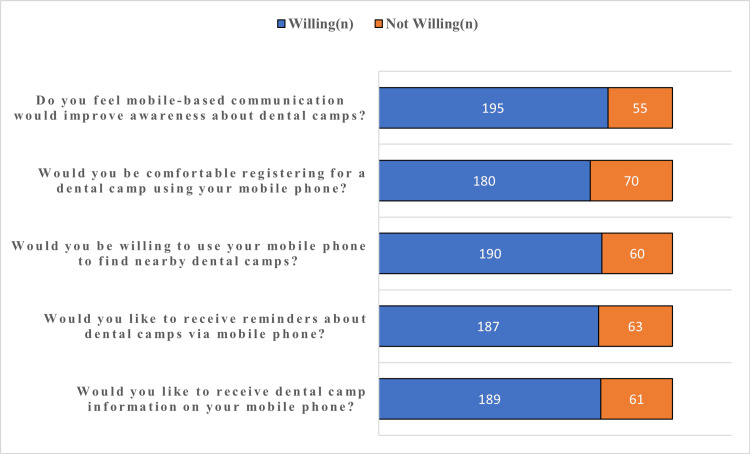
Perception toward mobile phone-based communication for dental outreach programs among study participants (n = 250)

Participants demonstrated a predominantly positive perception toward mobile phone-based communication for dental outreach programs (Figure 3). Willingness to receive dental outreach camp information (75.6%), receive reminders (74.8%), and locate nearby dental camps (76.0%) via mobile phones was high. It should be noted that these figures reflect willingness exclusively among participants who were already smartphone users, as the QR code-based recruitment method structurally excluded individuals without smartphone access. These estimates may therefore overrepresent mobile communication acceptance in the broader outreach attendee population. Furthermore, 72.0% of participants expressed comfort with registering for dental camps using mobile phones, and 78.0% believed that mobile-based communication would improve awareness regarding dental outreach programs.

**Figure 4:**
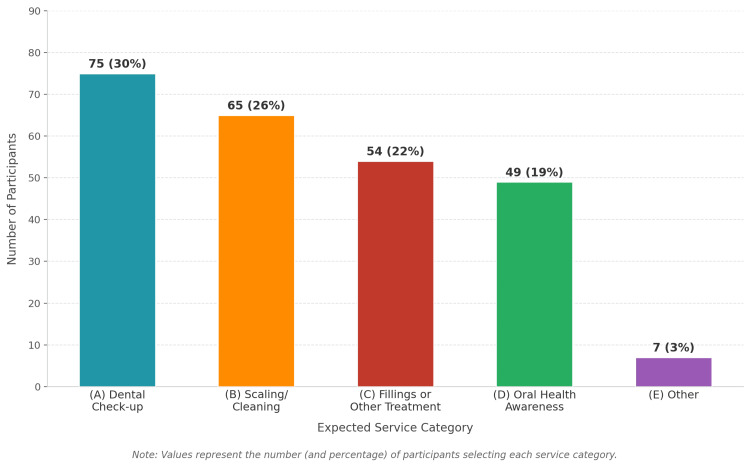
Expectations regarding services provided during dental outreach camps among study participants (multiple responses permitted; n = 250). Values represent the percentage of participants who selected each service category

Regarding expectations of services provided during dental outreach camps, dental check-ups (80.0%) and scaling or cleaning (70.0%) were the most commonly expected services, followed by oral health education (58.0%) and fillings or other dental treatments (52.0%) (Figure 4).

The association between age group and willingness toward mobile phone-based communication for dental outreach programs (Table 2), in which statistically significant associations were observed between age group and willingness to receive dental outreach camp information via mobile phone (p = 0.003*), willingness to receive reminders (p = 0.002*), comfort with registering for dental camps using mobile phones (p = 0.045), and perception that mobile-based communication would improve awareness (p = 0.001*). However, no statistically significant association was found between age group and willingness to use mobile phones to locate nearby dental camps (p = 0.073). Overall, younger age groups demonstrated greater willingness and acceptance of mobile phone-based communication compared to older participants.

**Table 2:** Statistical analysis was performed using Fisher’s exact test where expected cell counts were <5. Test statistics and exact p-values are reported. A p-value of <0.05 was considered statistically significant

S. no	Question	Age group	Willing n (%)	Not willing n (%)	χ² value	p-value
1	Receive dental camp information on your mobile phone	18-25	60 (81.1)	14 (18.9)	16.5426	0.003*
		26-40	84 (77.8)	24 (22.2)		
		41-60	31 (62.0)	19 (38.0)		
		>60	14 (77.8)	4 (22.2)		
2	Receive reminders about dental camps via a mobile phone	18-25	59 (79.7)	15 (20.3)	14.2053	0.002*
		26-40	82 (75.9)	26 (24.1)		
		41-60	32 (64.0)	18 (36.0)		
		>60	14 (77.8)	4 (22.2)		
3	Use a mobile phone to find nearby dental camps	18-25	61 (82.4)	13 (17.6)	15.9540	0.073
		26-40	84 (77.8)	24 (22.2)		
		41-60	32 (64.0)	18 (36.0)		
		>60	13 (72.2)	5 (27.8)		
4	Comfortable registering for the dental camp using a mobile phone	18-25	55 (74.3)	19 (25.7)	13.5655	0.045*
		26-40	76 (70.4)	32 (29.6)		
		41-60	30 (60.0)	20 (40.0)		
		>60	14 (77.8)	4 (22.2)		
5	Mobile-based communication improves awareness	18-25	60 (81.1)	14 (18.9)	15.3379	0.001*
		26-40	87 (80.6)	21 (19.4)		
		41-60	30 (60.0)	20 (40.0)		
		>60	18 (100.0)	0 (0.0)		

## Discussion

Our study demonstrates that word-of-mouth emerged as the primary awareness channel for dental outreach camps among Chennai attendees (66.8%). Kakatkar et al. [3], studying barriers to dental service utilization in Udaipur, similarly identified interpersonal communication and social networks as the dominant source of health information among rural populations, with print materials achieving limited penetration, reflecting literacy-linked contextual differences. A meta-analysis of dental care utilization among the Indian adult population by Talukdar et al. [4] further corroborates that awareness and information-related factors remain the primary determinants of dental service uptake across diverse Indian settings, with advance notification and structured communication significantly improving service attendance rates.

Gambhir et al. [5], in their comprehensive review of dental care utilization patterns across India, highlighted that in urban and semi-urban populations, dependence on informal dissemination remains high despite relatively greater literacy levels, with poster and print-based communication contributing modestly to awareness. This pattern is consistent with the negligible print visibility (5.2%) and complete absence of social media uptake (0%) observed in the present study, despite substantially higher literacy levels among our participants (74.8% with secondary education or above). These findings suggest that neither literacy alone nor the presence of conventional information channels is sufficient to ensure timely advance awareness of dental outreach camps in rapidly urbanizing settings such as Chennai [4].

Pre-camp awareness of available services was limited in our study (36.0%). Participant ratings for information clarity (32.8%) and location convenience (42.0%) were likewise inferior to the 65-70% satisfaction levels reported in rural outreach settings by Shrestha et al. [6], a disparity plausibly linked to traffic congestion and transport constraints characteristic of semi-urban Chennai [5]. Same-day awareness in our cohort (59.2%) underscores persistent dissemination gaps that disproportionately affect spontaneous, low-income attendees in Tamil Nadu's suburban zones, consistent with utilization patterns documented across Indian outreach programs [3,4].

Acceptance of mobile-based communication in our cohort was notably high (75.6%). DeSouza et al. [7], in a seminal study of mobile phone acceptability for healthcare delivery in rural India, found that a substantial majority of participants expressed willingness to receive health-related information via mobile platforms. This is further supported by studies demonstrating the effectiveness of SMS-based oral health education in Indian populations [8,9]. The high acceptance observed in the present study reflects Tamil Nadu's high smartphone penetration, particularly among the 18-40 age group, who comprised 72.8% of our sample, and highlights the feasibility of integrating mobile-based communication into dental outreach program planning in urban and semi-urban settings.

Age-stratified analysis mirrored national trends in digital health adoption. Young adults aged 18-25 years demonstrated the highest acceptance (81.1%), followed by a significant decline among participants aged 41-60 years (62.0%; p = 0.003), consistent with digital health challenges and technology hesitancy described for older adult populations in India by Gudi et al. [10]. Interestingly, participants older than 60 years (n = 18) showed unexpectedly high acceptance levels (77.8-100%). This may reflect selective participation by technologically proficient older adults who were more comfortable operating smartphones; however, given the very small subsample size, this observation must be interpreted with considerable caution. It should be regarded as a hypothesis for future investigation rather than a definitive finding, and conclusions regarding mobile communication acceptance among older adults should not be drawn from this data alone.

Service preferences in our study, particularly for routine dental check-ups (80%) and scaling (70%), aligned with preventive care priorities emphasized in the National Oral Health Programme [11] and with the WHO Global Strategy on Oral Health [12], which emphasize scaling up preventive and promotive oral health services at the community level. Restorative treatment interest in our cohort (52%) exceeded the extraction-dominant patterns reported in urban populations by Gambhir et al. [5], suggesting a gradual shift toward preventive care awareness among urban outreach attendees in Tamil Nadu.

Overall, these findings reveal a coexistence of entrenched traditional awareness pathways with growing digital receptivity, supporting hybrid outreach strategies that integrate youth-focused digital communication with the Accredited Social Health Activist (ASHA)-mediated interpersonal communication for older adults. Such approaches may enhance advanced awareness beyond 60%, in line with National Oral Health Policy objectives [2] and National Oral Health Programme goals [11]. Evaluations assessing downstream service utilization gains are essential to translate cross-sectional observations into scalable, equity-oriented interventions, as emphasized by Kadaluru et al. [13] in their study on oral health care utilization at community outreach programs in South India.

Limitations and future directions

This cross-sectional study limits causal inferences between awareness pathways and willingness toward mobile phone-based communication. As participation required smartphone access and completion of a QR code-based survey, acceptance of mobile-based communication may be overestimated. The study included only dental outreach camp attendees, which may limit the representativeness of the wider community. Responses were self-reported and may be subject to recall and social desirability bias. The small number of participants aged above 60 years and the restriction to selected rural and semi-urban areas of Chennai further limit generalizability. Furthermore, content validity was assessed using a panel of three expert reviewers, while the CVR of 0.84 indicates good validity, a larger expert panel would have further strengthened the robustness of this estimate, as Lawshe's method is ideally applied with panels of greater size. Additionally, the study did not employ multivariate analysis; therefore, the independent effect of age on willingness toward mobile phone-based communication, after controlling for potential confounders such as educational level and occupation, could not be determined. Future studies should consider logistic regression modelling to account for such confounding. Finally, the smartphone-based recruitment approach, while practical for a camp setting, structurally excluded individuals without smartphone access, which likely resulted in an overestimation of mobile communication acceptance and should be considered when interpreting the study's primary findings. Future studies will employ longitudinal or interventional designs to assess the impact of mobile-based communication on advanced awareness and service utilization. Evaluating such hybrid models across diverse settings will help inform scalable and equitable oral health outreach strategies aligned with National Oral Health Policy goals [2].

## Conclusions

Overall, this study assessed awareness of dental outreach camps and willingness toward mobile phone-based communication among attendees in Chennai. Awareness was predominantly interpersonal (66.8%), with limited advance knowledge of services (36.0%) and low information clarity ratings (32.8%). Most participants expected preventive and basic treatment services, and 75.6% of smartphone-owning participants expressed willingness to receive outreach-related information through mobile phone-based communication, with younger age groups showing significantly greater acceptance, though these estimates should be interpreted in light of the smartphone-based sampling approach employed. These findings underscore the need to bridge existing information dissemination gaps by integrating mobile phone-based strategies with community-based approaches to enhance awareness and utilization of dental outreach camps. Future interventional studies are warranted to evaluate the impact of such hybrid communication models on oral health service uptake across diverse settings.
